# Thoracic extramedullary hematopoiesis: a rare clinical image

**DOI:** 10.11604/pamj.2024.47.132.43083

**Published:** 2024-03-22

**Authors:** Ashwin Karnan, Anjana Ledwani

**Affiliations:** 1 Department of Respiratory Medicine, Datta Meghe Institute of Higher Education and Research, Sawangi (Meghe), Wardha, Maharashtra, India

**Keywords:** Anemia, thalassaemia, erythropoiesis, splenectomy

## Image in medicine

A 22-year-old male presented to the outpatient department with complaints of fever, cough with expectoration, and breathlessness for the past 8 days. The patient is a known case of thalassaemia major with a history of splenectomy 10 years back. Chest X-ray showed bilateral homogenous shadows with suspicion of eosinophilic pneumonia, septic emboli, or metastases to the lung. Computed tomography of the thorax showed multiple well-defined para-osseous soft tissue density masses suggestive of thoracic extramedullary hematopoiesis. Chronic anemia may lead to extramedullary hematopoiesis due to failure of erythropoiesis in the bone marrow. Etiology includes myeloproliferative disorders and hemoglobinopathies. These masses are usually hypervascular and fine needle aspiration is preferred over biopsy. Treatment modalities include excision, radiotherapy, and repeated blood transfusion to decrease extramedullary hematopoiesis.

**Figure 1:**
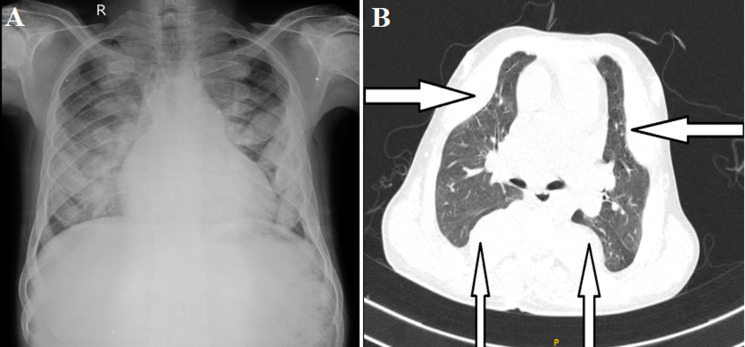
A) chest X-ray of the patient showing homogenous opacities with splaying of the ribs bilaterally; B) computed tomography of the thorax with white arrow showing sites of extramedullary hematopoiesis

